# How can psychosomatic physicians contribute to behavioral medicine?

**DOI:** 10.1186/s13030-016-0060-x

**Published:** 2016-03-03

**Authors:** Kazuhiro Yoshiuchi

**Affiliations:** Department of Stress Sciences and Psychosomatic Medicine, Graduate School of Medicine, The University of Tokyo, 7-3-1 Hongo Bunkyo-ku, Tokyo, Japan

## Abstract

In Japan, there is a unique clinical department, “Psychosomatic Medicine”, while there is not a department of behavioral science or behavioral medicine in medical schools. Although only eight medical schools have the department, psychosomatic physicians in the department have been involved with behavioral medicine. In the present manuscript, the author would like to introduce the contribution to behavioral medicine made by psychosomatic physicians in three aspects, education, clinical settings, and research, and propose some strategy for psychosomatic physicians to get more involved with behavioral medicine.

## Background

In Japan, there is not a department of behavioral science or behavioral medicine in medical schools. Instead, there is a unique clinical department, The Department of Psychosomatic Medicine. However, only eight medical schools have the department.

Medical doctors in the department are basically internists who treat patients with stress-related diseases. They can use both pharmacology and psychotherapy, which means that they are familiar with behavior modification.

Therefore, psychosomatic medicine in Japan is very closely associated with behavioral medicine. In fact, all of the board members of the clinical section of the Japanese Society of Behavioral Medicine are psychosomatic physicians, and medical doctors in the department (psychosomatic physicians) also play an important role in the medical education for behavioral medicine in the eight medical schools. I would like to introduce three aspects in behavioral medicine that are closely associated with psychosomatic physicians in Japan.

### Education

First, I would like to introduce the current status of education for behavioral medicine in medical schools by departments of psychosomatic medicine [1]. In Japan, medical schools have a six-year course, which students can enter directly after graduating from high school. In the eight medical schools, every department of psychosomatic medicine is involved with “psychosomatic medicine” as a part of internal medicine for the 4th-year medical student. In some medical schools, psychosomatic physicians are involved with problem-based learning classes.

In addition, they are involved with clinical clerkship for the 5th-year medical students for one week or two weeks. In some medical schools, half of the students experience the department of psychosomatic medicine during their clinical clerkship, while all of the students experience the department of psychosomatic medicine in the other medical schools. Psychosomatic physicians in the eight medical schools also teach the 5th-year or 6th-year medical students for their elective clinical clerkship for four weeks.

Table 1 shows the themes for the course of “psychosomatic medicine” for the 4th-year medical students in The University of Tokyo. They include the stress model in behavioral medicine, some psychotherapies, and behavior modification techniques such as the transtheoretical model and empowerment, which are closely associated with behavioral medicine.

**Table 1:** Themes for the course of psychosomatic medicine as part of internal medicine at The University of Tokyo

overview of psychosomatic medicine
non-pharmacological treatment in psychosomatic medicine
cardiovascular diseases and respiratory diseases influenced by psychosocial factors
neuromusculoskeletal diseases influenced by psychosocial factors
eating disorders
diabetes and behavioral modification

Some departments of psychosomatic medicine in the eight medical schools also hold an open seminar for medical students, young doctors, clinical psychologists, and nurses. The seminars also include many themes in behavioral medicine.

### Clinical settings

In Japan, department of psychosomatic medicine was originally established in order to see patients with “psychosomatic diseases”. “Psychosomatic diseases” are defined by the Japanese Society of Psychosomatic Medicine as physical diseases that are closely influenced by psychosocial factors in terms of their onset/course and that are not merely physical symptoms caused by psychiatric disorders [2]. The Japanese Society of Psychosomatic Medicine also stated that psychosomatic medicine was closely associated with behavioral medicine in the clinical guidelines published in 1991 [2]. Therefore, “psychosomatic diseases” can be applied with the “stress model” in behavioral medicine. The following diseases can be considered frequently as psychosomatic diseases: coronary artery diseases, essential hypertension, bronchial asthma, irritable bowel syndrome, functional dyspepsia, Graves’ disease, diabetes, tension-type headache, and migraine.

In addition, behavioral medicine is included in the curriculum for becoming Board Certified Psychosomatic Internists. Psychosomatic physicians in Japan are required to be able to apply non-pharmacological intervention such as psychotherapies and behavioral modification as well as pharmacological intervention. Cognitive behavioral therapy, transactional analysis, and autogenic training are considered the three essential psychotherapies for psychosomatic physicians in Japan [3]. Therefore, psychosomatic physicians use intervention developed in behavioral medicine.

### Research

**Fig. 1:**
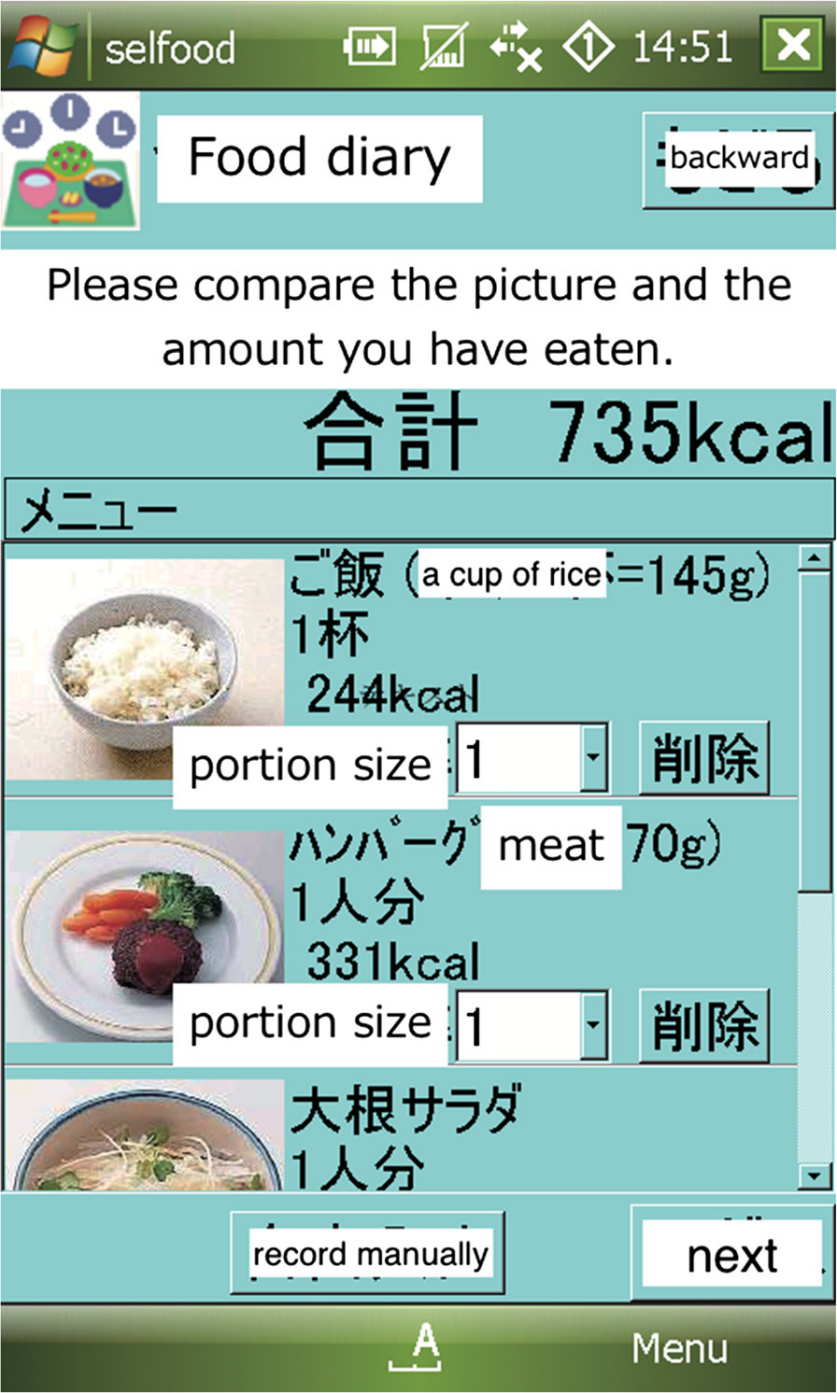
The electronic food diary that we have developed includes a photo menu of food items in order to let users determine the potion size of each food item easily. First, users choose a food or drink item from the menu list. Then, they choose the portion size from the list “0.25, 0.5, 0.75, 1, 1.25, 1.5, 2, 2.5, 3, 3.5, 4, 5” judging from the picture shown on the screen. If users have nutrient information about their meal, they are also able to record it manually into the food diary

Many studies in behavioral medicine have been conducted by psychosomatic physicians in Japan. For example, I and my colleagues have been involved in research using the ecological momentary assessment (EMA) method, which was originally developed by Stone and Shiffman in behavioral medicine. We first demonstrated that traditional assessment of headache intensity based on patient recall was not reliable using EMA [4]. Then, we extended measurement variables to locomotor activity, which are an objective variable, in addition to subjective symptoms. [5] Our recent study showed that momentary mood states might be predicted by locomotor activity in natural settings [6]. In addition, we are trying to develop an ecological momentary intervention (EMI) system, which uses data collected by EMA for treatment in natural settings. First, we developed an electronic food diary (Fig. 1) for which the accuracy was validated with evaluation by registered dietitians [7]. Then, we extended the function of the electronic food diary to show graphs of intake calories momentarily with an individuals’ target calorie as a negative feedback system, although the efficacy of the system remains to be confirmed [8].

## Conclusion

Psychosomatic physicians in Japan have been involved with and contributed to the field of behavioral medicine. However, these activities have not been acknowledged by medical doctors in other areas and general people. Therefore, psychosomatic physicians should interact with doctors in other areas and get involved with publicity activities for spreading the role of psychosomatic physicians in behavioral medicine.

## Abbreviations

EMA: ecological momentary assessment
EMI: ecological momentary intervention
